# Current gaps and ideological trends in physician understanding of LGBTQ+ healthcare and its delivery in London, Ontario

**DOI:** 10.15694/mep.2021.000179.1

**Published:** 2021-06-28

**Authors:** Rachelle Beanlands, Lilian Robinson, Teresa Van Deven

**Affiliations:** 1 University of Ottawa; 2 University of Toronto; 3 Western University

**Keywords:** LGBTQ+ health, cultural competence, health equity, medical education, physician knowledge

## Abstract

This article was migrated. The article was marked as recommended.

Purpose

Lesbian, gay, bisexual, transgender, and queer (LGBTQ+) communities have unique healthcare needs that often go unmet. LGBTQ+ patients report higher levels of satisfaction with and are more likely to seek care from providers who possess knowledge of and demonstrate comfort with LGBTQ+ healthcare issues. We sought to determine knowledge of, comfort with, and perceived barriers to providing equitable LGBTQ+ care amongst physicians in London, ON.

Methods

Anonymous online surveys were distributed to roughly 2400 full- and part-time physicians at the Schulich School of Medicine & Dentistry. Co-investigators independently coded forty-two surveys and conducted a theoretical thematic analysis.

Results

Physicians were categorized according to their beliefs about the unique health needs of LGBTQ+ populations and the degree to which they possessed corresponding knowledge. Seventeen physicians (42%) believed that LGBTQ+ populations have unique needs and possessed knowledge, sixteen (38%) believed that LGBTQ+ populations have unique needs but lacked knowledge, and nine (21%) denied the existence of unique needs. Across all respondents, competence was lacking in three domains: transgender healthcare, responding to LGBTQ+ identity disclosure, and knowledge of systemic inequities faced by LGBTQ+ communities.

Conclusions

These findings elucidate knowledge gaps amongst a representative sample of physicians and present opportunities for targeted educational intervention to improve LGBTQ+ care.

## Introduction

Several documented health disparities exist between members of the lesbian, gay, bisexual, transgender and queer (LGBTQ+) communities and their heterosexual, cisgender counterparts. LGBTQ+ populations exhibit higher rates of mood disorders, completed suicide ( Mustanski, Garofalo and Emerson, 2010; Lehavot and Simoni, 2011; Kessler *et al.*, 2012; Nock *et al.*, 2013; Pakula and Shoveller, 2013; Russell and Fish, 2016), tobacco use ( King, Dube and Tynan, 2012), and illicit drug consumption ( Kecoievic *et al.*, 2012). Young LGB individuals have also been shown to have elevations in markers of cardiovascular stress ( Hatzenbuehler, McLaughlin and Slopen, 2013), lesbian and bisexual women tend to have higher BMI and rates of alcohol misuse than straight women ( Case *et al.*, 2004; Boehmer, Bowen and Bauer, 2007; Lehavot and Simoni, 2011), and gay and bisexual men are at greater risk for specific cancers and sexually transmitted infections than heterosexual men ( Jeffries *et al.*, 2012; Poynten and Grulich, 2013). In addition, LGBTQ+ individuals also have various unmet health needs, including preventative care and cancer screening, as well as access to medical transition in the setting of increasing demand for gender-affirming care ( Facione and Facione, 2007; Bauer, 2010; Giblon and Bauer, 2017; Bell and Purkey, 2019).

In attempting to explain these disparities, it is useful to apply minority stress theory, stipulating that both external and internal forces play a part in producing chronic stress amongst minority populations ( Meyer, 1995; Meyer, 2003). In turn, chronic stress directly impacts upon health outcomes, and may lead to service avoidance as well as high-risk health behaviours. Stigma, whether experienced, anticipated, or internalized by patients, is a strong predictor of decreased healthcare utilization ( Daley and MacDonnell, 2011; Whitehead, Shaver and Stephenson, 2016). In fact, a single negative interaction with the healthcare system has been shown to tarnish all future interactions for LGBTQ+ patients, resulting in service avoidance ( Rounds, McGrath and Walsh, 2013). There are many ways in which a provider-patient interaction can be negative for LGBTQ+ individuals. For example, a negative encounter may be characterized by providers making assumptions about sexual orientation and gender identity, displaying discomfort with topics surrounding sexuality or gender, lacking knowledge about diverse sexual practices, or responding insensitively to disclosures of gender identity or sexual orientation ( Kosenko *et al.*, 2013; Baldwin *et al.*, 2016; Bell and Purkey, 2019). Some interactions can even involve refusal of care and overt discrimination ( Kosenko *et al.*, 2013).

To better meet the needs of LGBTQ+ patients, healthcare delivery must be optimized to address existing disparities. Studies have shown that LGBTQ+ care can be improved upon in a variety of ways. For instance, LGBTQ+ patients are more likely to be satisfied with their healthcare and follow-up with providers more routinely when they are able to speak openly about sexual orientation, gender identity, sexual activity, and high risk behaviors during appointments ( Hoffman, Freeman and Swann, 2009; Mosack, Brouwer and Petroll, 2013; Rounds, McGrath and Walsh, 2013; Baldwin *et al.*, 2016; Bell and Purkey, 2019). Importantly, disclosure of sexual orientation and/or gender identity can be especially daunting for individuals in a healthcare setting due to the power differential inherent to patient-provider relationships and the fear of diminished quality of care as a result of disclosure ( Kosenko *et al.*, 2013; Whitehead, Shaver and Stephenson, 2016; Bell and Purkey, 2019). It is therefore critical that providers foster an environment that allows for patients to safely disclose their identity. Survey data shows that LGBTQ+ patients are more likely to come out to providers who have knowledge of LGBTQ+ issues, avoid assumptions in history-taking, are comfortable discussing non-normative identities and lifestyles ( Hoffman, Freeman and Swann, 2009; Rounds, McGrath and Walsh, 2013; Baldwin *et al*., 2016), and can either provide or refer patients to services including hormone therapy and gender-affirming care ( Coleman *et al.*, 2012; Bell and Purkey, 2019).

Despite knowledge of how care is experienced by LGBTQ+ patients, gaps exist in the literature pertaining to physician perspectives on providing care to LGBTQ+ patients ( Knight *et al.*, 2014). Knowledge of these perspectives may help to inform policymakers, educators, and individual healthcare providers in their attempts to address health disparities experienced by LGBTQ+ communities. In this study, we seek to understand physician knowledge of LGBTQ+ health needs, as well as the personal and institutional challenges faced by physicians when caring for these populations. We surveyed physicians in London, Ontario to assess their knowledge of LGBTQ+-specific health issues, ideas about the state of LGBTQ+ healthcare, comfort level with LGBTQ+ patients, and willingness to undergo cultural competency training.

## Methods

### Data Collection

We implemented The Checklist for Reporting Results of Internet E-Surveys (CHERRIES) for the design and analysis of surveys ( Eysenbach, 2004), and generated anonymous online surveys using Qualtrics. Usability and technical functionality were tested by co-investigators prior to distribution. The Principal Investigator distributed a permanent hyperlink via email to a convenience sample of roughly 2,400 full- and part-time faculty members at the Schulich School of Medicine and Dentistry. Prior to survey initiation, respondents were required to read an electronic Letter of Information, and consent was implied by survey completion. The survey link was distributed only once, and remained open for one month. We provided no monetary compensation for study participation. Surveys consisted of a demographics section, four Likert-scaled questions and five long-answer questions. The complete survey is available as Supplementary File 1. No items were mandatory for completion, and respondents were able to review and change their answers before submission.

### Participant Eligibility

To be included in this study, physicians were required to practice in London, Ontario and be a faculty member at the Schulich School of Medicine and Dentistry. We considered surveys incomplete if one or more long-answer questions were not responded to, and excluded incomplete survey responses from this study. Qualtrics software assigned each participant a unique IP address corresponding to the client computer, and we reviewed IP addresses to limit each respondent to one unique submission. We did not identify any duplicate entries.

### Data Analysis

In total, 54 surveys were submitted. Of those, we found 12 responses to be incomplete and excluded these responses from data analysis. We independently coded a total of 42 surveys and subjected coded surveys to a theoretical thematic analysis. We assigned each respondent a unique alphanumeric code indicating their response number as designated by Qualtrics-generated timestamp, type of practice, and sexual orientation. We coded type of practice as specialist (A), family physician (B), or unspecified (X), and sexual orientation as queer (q), heterosexual (h), or unspecified (x). For example, a heterosexual family physician who was the first survey respondent would be identified by the following code: 1Bh. We assigned scaled responses a numerical value and analyzed across all respondents to identify quantitative trends, and triangulated thematic findings with existing literature to compare and contrast themes.

## Results/Analysis

### Respondent Demographics

Seventy-one unique site visitors viewed the Letter of Information. Amongst unique site visitors, 60 (85%) agreed to participate in the study, and of those who agreed to participate, 42 (70%) completed the survey. Of the 42 respondents who completed the survey, 24 (57%) were male, 17 (40%) female, and 1 (2%) identified as non-binary. The majority (n= 31, 74%) identified as heterosexual, 8 (19%) as gay, lesbian, bisexual, or queer, and 3 (7%) did not provide a response. Most respondents (n = 36, 86%) were between the ages of 35 and 64, practiced as a specialist (n = 36, 86%), and worked in an academic center (n = 35, 83%). Years in practice varied widely, with 18 (43%) of the respondents having been in practice for 20 years or less, and 24 (57%) for more than 20 years. Most respondents were Caucasian (n = 35, 83%), South Asian (n = 3, 7%), or an ethnicity not included in the options provided (n = 3, 7%) ( Table 1).

**Table 1:** Demographic data of physician respondents

Individual-Level Demographic Variables	Number of Physicians	Percentage of Physicians
AGE		
25-34	2	4.76
35-44	14	33.33
45-54	10	23.81
55-64	12	28.57
65+	4	9.52
GENDER		
Male	24	57.14
Female	17	40.48
Non-Binary	1	2.38
SEXUAL ORIENTATION		
Heterosexual	31	73.81
Gay	3	7.14
Lesbian	2	4.76
Bisexual	2	4.76
Misunderstood Question	2	4.76
Queer	1	2.38
Didn't Say	1	2.38
ETHNICITY		
White	35	83.33
South Asian	3	7.14
Other	3	7.14
East Asian	1	2.38
Black	0	0.00
Arab	0	0.00
Indigenous	0	0.00
YEARS IN PRACTICE		
1-5	1	2.38
6-10	10	23.81
11-15	4	9.52
16-20	3	7.14
21-25	10	23.81
25+	14	33.33
SPECIALTY		
Specialist	36	85.71
Family Physician	5	11.9
Didn't Say	1	2.38
PLACE OF PRACTICE		
Academic Centre	35	83.33
Community Clinic	6	14.29
Mental Health Facility	1	2.38

### Physician Categorization

Physicians were categorized into one of three primary categories according to their beliefs about the unique health needs of LGBTQ+ populations and the degree to which they possessed knowledge about said needs. Seventeen physicians (42%) - positive respondents - believed that LGBTQ+ individuals have unique needs and presented considerable knowledge to substantiate that claim. Sixteen physicians (38%) - intermediate respondents - believed that LGBTQ+ individuals have unique health needs but lacked knowledge about them. Nine physicians (21%) - negative respondents - believed that LGBTQ+ patients do not have unique health needs and lacked knowledge about LGBTQ+ care. Physicians were secondarily categorized according to the nature of their remaining survey responses. The majority of positive and intermediate respondents were globally positive in their regard for LGBTQ+ populations, while the majority of negative respondents were globally negative ( Table 2).

**Table 2:** Secondary categorization of positive, intermediate, and negative physician respondents

	Number of Physicians in Secondary Category (% of Those from Primary Category)			
Primary Physician Category	Globally Positive *	Globally Negative **	Neither ***	Total
**Positive**	**12 (71%)**	**1 (6%)**	**4 (23%)**	**17**
**Intermediate**	**8 (50%)**	**5 (31%)**	**3 (19%)**	**16**
**Negative**	**0 (0%)**	**7 (78%)**	**2 (22%)**	**9**

* *Global positive regard was denoted by recognition of both systemic inequities and barriers to the provision of equitable care, as well as demonstrationof allyship through support for coming out or seeking to identify and fill individual knowledge gaps*

** *Global negative regard was denoted by an expressed belief that LGBTQ+ healthcare is equitable and that no barriers exist to the provision of care, as well as demonstration of a lack of support for LGBTQ+ populations through minimization of identity or explicit discrimination.*

*** *Physicians were considered neither globally-positive or globally-negative if they did not satisfy all respective criteria*

**Figure 1:**
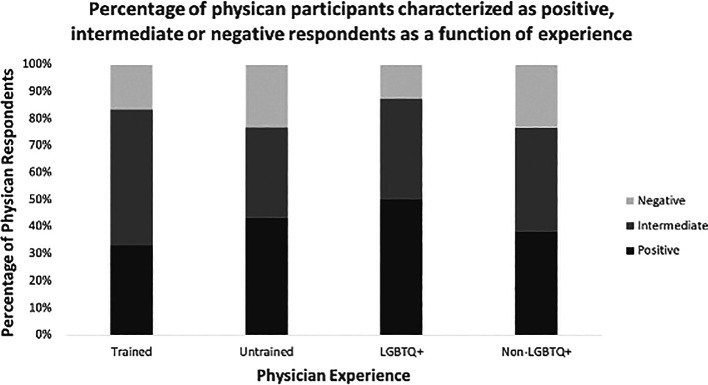
Percentage of physician participants characterized as positive, intermediate or negative respondents as a function of experience.

Categorization of respondents as positive, intermediate, or negative was predicted by whether or not respondents possessed lived and/or acquired experience in LGBTQ+ care ( Figure 1).

In general, LGBTQ+ physicians possessed greater knowledge of health inequities and were more supportive of LGBTQ+ patients than non-LGBTQ+ physicians. Similarly, physicians with acquired training in LGBTQ+ health were less likely to be categorized as negative in their responses than their untrained counterparts ( Table 3).

**Table 3:** Acquired or lived experience level of physician respondents

	Experience Level (% of Physicians According to Primary Category)			
Primary Physician Category	Trained (%)	Untrained (%)	LGBTQ+ Identifying (%)	Non-LGBTQ+ Identifying (%)
**Positive**	**4 (33.3%)**	**13 (43.3%)**	**4 (50%)**	**13 (38.2%)**
**Intermediate**	**6 (50%)**	**10 (33.3%)**	**3 (37.5%)**	**13 (38.2%)**
**Negative**	**2 (16.7%)**	**7 (23.3%)**	**1 (12.5%)**	**8 (23.5%)**
**Total**	**12 (100%)**	**30 (100%)**	**8 (100%)**	**34 (100%)**

### Likert Scale Semi-Quantitative Data

Likert scaled responses to four survey questions revealed that respondents in the intermediate category felt most positive about their interactions with LGBTQ+ patients, were most comfortable with LGBTQ+ identity disclosure, and, on average, agreed more strongly that they possessed enough knowledge to care for LGBTQ+ patients. Positive respondents were more likely than intermediate respondents to believe that LGBTQ+ patients are discriminated against. Negative respondents felt the least comfortable with LGBTQ+ patients, and thought they were rarely discriminated against ( Table 4).

**Table 4 (A-D):** Likert-scale responses from physician respondents

**Table 4A:** Scaled Question One: How would you rate your interactions with LGBTQ+ patients?

	Number of Physicians by Primary Category (% of Physicians)		
Scaled Response	Positive	Intermediate	Negative
**Strongly Positive**	**2 (11.8%)**	**8 (50%)**	**2 (22.2%)**
**Positive**	**13 (76.5%)**	**4 (25%)**	**4 (44.4%)**
**Neither Positive nor Negative**	**1 (5.9%)**	**4 (25%)**	**3 (33.3%)**
**Somewhat Negative**	**-**	**-**	**-**
**Strongly Negative**	**-**	**-**	**-**
**Didn't Respond**	**1 (5.9%)**	**-**	**-**
**Total**	**17 (100%)**	**16 (100%)**	**9 (100%)**

**Table 4B:** Scaled Question Two: How comfortable are you with disclosure?

	Number of Physicians by Primary Category (% of Physicians)		
Scaled Response	Positive	Intermediate	Negative
**Very Comfortable**	**9 (53%)**	**14 (87.5%)**	**5 (55.6%)**
**Somewhat Comfortable**	**7 (41.2%)**	**1 (6.3%)**	**2 (22.2%)**
**Neither Comfortable nor Uncomfortable**	**-**	**1 (6.3%)**	**2 (22.2%)**
**Somewhat Uncomfortable**	**-**	**-**	**-**
**Very Uncomfortable**	**-**	**-**	**-**
**Didn't Respond**	**1 (5.9%)**	**-**	**-**
**Total**	**17 (100%)**	**16 (100%)**	**9 (100%)**

**Table 4C:** Scaled Question Three: How often are LGBTQ+ discriminated against?

	Number of Physicians by Primary Category (% of Physicians)		
Scaled Response	Positive	Intermediate	Negative
**Always**	**1 (5.9%)**	**1 (6.3%)**	**-**
**Often**	**1 (5.9%)**	**5 (31.3%)**	**-**
**Sometimes**	**14 (82.4%)**	**5 (31.3%)**	**-**
**Rarely**	**-**	**2 (12.5%)**	**7 (77.8%)**
**Never**	**-**	**2 (12.5%)**	**2 (22.2%)**
**Didn't Respond**	**1 (5.9%)**	**1 (6.3%)**	**-**
**Total**	**17 (100%)**	**16 (100%)**	**9 (100%)**

**Table 4D:** Scaled Question Four: I possess enough knowledge to care for LGBTQ+ patients

	Number of Physicians by Category (% of Physicians)		
Scaled Response	Positive	Intermediate	Negative
**Strongly Agree**	**3 (17.6%)**	**6 (37.5%)**	**2 (22.2%)**
**Somewhat Agree**	**9 (53.0%)**	**8 (50%)**	**6 (66.7%)**
**Neither Agree nor Disagree**	**3 (17.6%)**	**1 (6.3%)**	**1 (11.1%)**
**Somewhat Disagree**	**1 (5.9%)**	**-**	**-**
**Strongly Disagree**	**-**	**1 (6.3%)**	**-**
**Didn't Respond**	**1 (5.9%)**	**-**	**-**
**Total**	**17 (100%)**	**16 (100%)**	**9 (100%)**

### Emerging Themes

Across all groups, several important themes emerged pertaining to prevalent ideologies, important gaps in knowledge, and mitigating factors that influence the delivery of LGBTQ+ healthcare by physicians. These themes included (1) a tendency to minimize the significance of LGBTQ+ identity disclosure, (2) the presence of tunnel vision amongst specialist physicians, and (3) identifying transcare as a black-box area of LGBTQ+ care.

Theme One: Minimization

Minimization was a theme amongst physician respondents and took two forms. First, respondents tended to minimize the significance of gender identity and/or sexual orientation disclosure by patients. While most demonstrated general acceptance of patients’ identities, they failed to acknowledge the difficulties patients may anticipate when coming out to them. For instance, when asked how they respond to a disclosure, one respondent did so positively but provided no insight into how they tailor their response to their LGBTQ+ patients:

Participant 30Ah -“Non judgmentally.”

Other participants were indifferent:

Participant 9Aq -“I don’t care. Doesn’t affect my area of care or provision of care”.

Several physicians regarded LGBTQ+ identity disclosure in the same way as they would the disclosure of any other medical information, in that physicians tended to acknowledge the importance of the information but failed to differentiate it from a patient’s chief complaint:

Participant 2Ah - “I treat it like it’s a fact that is important - just like I respond when someone tells me they have chest pain.”

Theme Two: Tunnel Vision

When asked to critically appraise the current state of LGBTQ+ healthcare, many physicians demonstrated an inability to extrapolate outside of their individual practice, an observation referred to here as ’tunnel vision’. This was more prevalent amongst specialists than primary care providers, with 61% of specialists and only 33% of family physicians demonstrating this tendency.

First, specialists in particular tended to believe that LGBTQ+ issues are not relevant to them in the context of their focused field of practice:

Participant 18Ah - “In the area of oncology I don’t believe that LGBTQ patients have any unique needs as compared to others.”

In addition, those who either denounced intolerance or indicated that they have observed only equitable care in their practice, tended to deduce that all LGBTQ+ care must therefore be equitable. For instance, when asked whether they felt that LGBTQ+ patients receive the same standard of care as their cis-gender heterosexual counterparts, one specialist said the following:

Participant 20Ax - “Yes. I have never seen discrimination [or] substandard care/inequitable care because of a person’s LGBTQ+ identifier.”

There was also a tendency amongst physicians to downplay their contribution to discrimination against LGBTQ+ patients. These respondents expressed that their interactions with LGBTQ+ patients are globally positive, and implied rather that other healthcare practitioners are responsible for the inequities faced by LGBTQ+ patients:

Participant 8Bh - “[...] I am limited in my influence over others on my team. my nurse, admin team and trainees are individuals on their own journey.”

When asked if LGBTQ+ patients receive equitable care in London, Ontario, another respondent stated:

Participant 25Aq -“I suspect they don’t [sic]. Some practitioners are not comfortable talking about gay issues.”

Theme Three: Transcare is a Black Box

Respondents across all groups demonstrated a lack of well-rounded knowledge about transgender healthcare. This was exemplified in several ways. First, when physicians alluded to the unique health needs of transgender individuals, they focused almost explicitly on transition care and rarely commented on longitudinal components of management such as ongoing endocrinological therapy and mental healthcare:

Participant 37Bq - “One of the challenges is supporting transgender patients at the start of their transition in terms of accessing the supports that they may require.”

A lack of overall knowledge was also exemplified by a fear of language. Physicians were wary of using improper pronouns, responding to disclosure, and discussing transgender issues:

Participant 16Ah - “It seems a bit like a moving target sometimes. Sometimes patients expect us to understand every variance in gender which is difficult because the experience is quite different from individual to individual.”

Respondents also expressed concerns about how the use of erroneous language might impact negatively upon the therapeutic relationship:

Participant 20Ax - “I fear being politically incorrect and saying something “wrong” by accident with trans patients, and ruining the patient-doctor relationship.”

Finally, physicians tended to focus on the unique needs of transgender individuals to the exclusion of other LGBQ+ groups. In many instances, this seemed to reflect the overemphasis of a topic that is both popular in the media and elusive to the average physician:

Participant 10Ah - “[m]ost gay men and women do not have unique health care needs. They are prone to the same diseases as everyone else. [...] Transsexual individuals who wish to transition do have unique healthcare needs.”

## Discussion

This study provides insight into the knowledge level of a representative sample of physicians and the current trends and gaps in their understanding of LGBTQ+ healthcare. We found that surveyed physicians could be categorized according to whether or not they believed that LGBTQ+ populations have unique health needs, and the extent of their knowledge corresponding to those needs. Importantly, physicians who believed that LGBTQ+ populations have unique needs and appeared to possess corresponding knowledge, were most likely to also recognize the existence of systemic healthcare inequities and provide explicit support for their LGBTQ+ patients. In contrast, those who believed that LGBTQ+ communities do not have unique needs and also lacked knowledge about LGBTQ+ healthcare, were more likely to demonstrate a global lack of knowledge about barriers to the provision of equitable care for LGBTQ+ patients, and even more likely to make discriminatory remarks. Within the study population, 59.5% of physicians lacked knowledge about LGBTQ+-specific needs, pointing to a significant knowledge gap amongst practicing physicians.

When we cross-referenced Likert-scale survey responses with physician categorization, several observations were made. Predictably, physicians who were least comfortable with LGBTQ+ patients according to their Likert-scale responses, were more likely to be categorized as ‘negative’ according to the nature of their narrative responses. Interestingly, physicians who were deemed to be most knowledgeable of LGBTQ+ issues - those categorized as ‘positive’ -, rated themselves as less comfortable providing care to LGBTQ+ patients than their ‘intermediate’ counterparts. One possible explanation for this observation is that as physicians acquire more education and experience, they gain self-awareness and better understand their individual limitations when providing care to a specific population. In keeping with this, ‘intermediate’ respondents indicated a high degree of comfort with LGBTQ+ issues on a Likert-scale but had less concrete evidence to defend their claims in the narrative portion of the survey.

Our findings not only demonstrate the existence of a significant knowledge gap amongst physicians, but also point to the role that acquired experience plays in narrowing this gap. Most notably, physicians with self-reported experience in LGBTQ+ care, whether obtained through lived experience or formal training, were more likely to have a globally-positive regard for their LGBTQ+ patients than their inexperienced counterparts. Existing literature tells us that physicians with greater knowledge of LGBTQ+ health will provide superior care to these groups ( Hoffman, Freeman and Swann, 2009; Rounds, McGrath and Walsh, 2013; Baldwin *et al*., 2016). These findings therefore lead us to suggest that more education leads to better care and that we should seek to fill specific knowledge gaps and correct commonly misheld beliefs through targeted educational interventions.

Across all respondents, we identified three opportunities for targeted education. These include (1) a lack of knowledge pertaining to transcare (2) minimization of LGBTQ+ identity disclosure in a clinical setting, and (3) the presence of ‘tunnel vision’, or a tendency amongst specialists to evade the presence of and downplay their individual contribution to systemic inequities faced by LGBTQ+ patients. Each of these trends present a unique opportunity for educational intervention. First, with respect to transcare, physician respondents lacked knowledge about trans health, conveyed a fear of language, and focused principally on transition to the exclusion of other elements of transcare. In the future, we propose that educators leverage data from Trans PULSE Canada, an ongoing national survey that is seeking to ascertain the lived experience of trans and non-binary Canadians ( Trans Pulse, 2019). Data from the Trans PULSE project will be used to inform gender-affirming policies and practices and should be integrated into medical curricula and continuing education in order to fill the gaps that were identified in our study. Resources such as Trans Care BC’s Gender Inclusive Language tool that serve to demystify gender-affirming language may also be helpful in reducing the fear experienced by physicians when communicating with trans and gender non-binary patients ( Trans Care BC, 2020). Second, given the observed tendency for a subset of physicians to minimize the importance of identity disclosure by LGBTQ+ patients, a critical conversation framework designed to delineate a person-centered approach to responding to a patient’s coming out, may be of benefit to physicians for whom this is of concern. Finally, to combat the existence of ‘tunnel vision’, medical students and practicing physicians should be made explicitly aware of specific inequities within the healthcare system, how they may passively and actively contribute as individual providers, and how they can best practice effective allyship to optimize the delivery of equitable care to LGBTQ+ patients.

Physician knowledge gaps identified in this study may be filled through the development and implementation of a targeted education tool. Although this was outside the scope of the present study, we intend to conduct further research to design and implement a cohesive resource for future and practicing physicians that will fill the specific gaps identified within this study. The proposed resource may take the shape of an online interactive module or seminar course, and should be informed by current educational theory, be mandatory for completion, and provide participants with concrete skills to implement when caring for LGBTQ+ patients. Ideally, the success of this resource will be measured through formal participant assessment and active engagement with LGBTQ+ stakeholders.

Notably, this study was subject to several limitations. First, response rate was limited and there was potential for response bias. Advertised as a study pertaining to LGBTQ+ care, it is possible that respondents were more likely to possess a positive attitude towards LGBTQ+ populations than those who elected not to respond. Furthermore, we acknowledge that although all attempts were made to analyze survey responses objectively, analysis may always be influenced by individual coder bias. Likewise, we may have misinterpreted survey responses or inadvertently taken them out of context. Finally, this study specifically aimed to understand existing gaps and barriers to the provision of LGBTQ+ healthcare as informed by and from the perspective of physicians. We therefore did not engage with LGBTQ+ community stakeholders and are drawing conclusions about stakeholder values on the basis of existing literature, only.

## Conclusion

This study adds to current LGBTQ+ health literature through the identification of specific knowledge gaps and ideological trends amongst a representative sample of practicing physicians. Given the existence and implications of these knowledge gaps, it is of critical importance that future and practicing physicians seek to identify and fill gaps in their practical and ideological understanding of LGBTQ+ health through individual research and/or formalized training in order to best deliver equitable and socially-accountable care.

## Take Home Messages

• More than half of surveyed physicians lacked knowledge about LGBTQ+ healthcare needs.
• Physicians with experience in LGBTQ+ care were more likely to feel positively about their LGBTQ+ patients.
• Areas identified for targeted education include care of transgender patients, physician response to LGBTQ+ identity disclosure, and knowledge of LGBTQ+ healthcare needs amongst specialists.
• Medical institutions may seek to fill these knowledge gaps by partnering with LGBTQ+ stakeholders to design and implement targeted education materials.

## Notes On Contributors

**Rachelle Beanlands:** Rachelle is a first year family medicine resident at the Univeristy of Ottawa. Rachelle has an interest in LGBTQ+ health, particularly transgender primary care, as well as adolescent medicine.

**Lilian Robinson:** Lilian is a first year family medicine resident at the University of Toronto. Lilian is interested in providing comprehensive primary and palliative care to LGBTQ+ and homeless populations.

**Teresa Van Deven:** Dr. Van Deven has worked in medical education for 14 years. She is currently the MD program faculty lead for Curriculum Oversight at Schulich School of Medicine & Dentistry. Teresa’s current focus is on implementing social medicine in undergraduate medical education (UGME) as well as national challenges of implementing competency-based UGME.
