# Supplementary material for: Current gaps and ideological trends in physician understanding of LGBTQ+ healthcare and its delivery in London, Ontario
Source: MedEdPublish (2016). 2021 Jun 28;10:179. [Version 1] doi: 10.15694/mep.2021.000179.1 (PMC10939580; doi:10.15694/mep.2021.000179.1)
Supplement: Peer review history

# Reviewer report: Reviewer response for version 1

**DOI:** 10.21956/mep.20439.r31659

**Published:** 2021-10-11

**Authors:** Keith Wilson

**Affiliations:** 1 Dalhousie University

This review has been migrated. The reviewer awarded 3 stars out of 5 The authors are to be commended for exploring this very important topic! Their mixed-method study included surveying full- and part-time physicians in the London, Ontario, Canada area. The introduction set up the need for this study very well.The quantitative data revealed a sample population of mostly Royal College specialists and those who mostly have been in practice for over 20 years. Surveys that were incomplete (from the open-ended questions perspective) were excluded from the analysis – although the survey software did not enforce required answers. About 2400 physicians were invited to participate, but only 60 responded and of those, and only 42 were considered complete (less than 2%). The authors noted that the email invite was only sent out once and this likely has influenced the number of responses. I disagree with the authors in concluding that this is a representative sample of physicians in that area – not only from the perspective of sample size but also of the Royal College/Family Medicine specialties split. Despite this, the authors present persuasive arguments that we certainly need more attention to this area of healthcare delivery.The open-ended questions were independently coded by two of the authors – the paper would be strengthened with more detail in how this analysis was conducted. The quotations from the participants were compelling examples for each of the themes that emerged. It would be helpful to know more detail about responses to a couple of questions from the survey that were not directly addressed in the article. In particular, the question about challenges faced by physicians may lend well to future directions that professional development could take. The discussion section was well written and the limitations they noted were well thought-out.Overall, this well-written article provides insight into the gaps that need to be addressed in LGBTQ+ healthcare provision. It should be widely read by those delivering care and involved in professional development even in the context of the caveats listed above.

Reviewer Expertise:

NA

No decision status is available

# Reviewer report: Reviewer response for version 1

**DOI:** 10.21956/mep.20439.r31658

**Published:** 2021-09-13

**Authors:** Anita Samuel

This review has been migrated. The reviewer awarded 4 stars out of 5 This is an informative study on an important topic. It is well-reported and the mixed methodology approach is effective in providing a deeper insight into the topic. The 2% response rate provides a very small sample making it difficult to generalize the findings from this study. It also raises questions of whether the survey should have been conducted over a longer duration and if reminders were sent to potential participants. Below are some suggestions to strengthen this article:• There are 2 interesting questions in the survey that are not reported in the article: challenges faced by the physicians and the percentage of LGBTQ patients they have worked with. The stated aim of this study was to understand personal and institutional challenges. But this is not sufficiently addressed in the article. • It might be interesting to know if increased experience working with LGBTQ+ patients influenced the responses. • In the results you note that physicians focus more on trans care and exclude other LGB groups. In your discussion section when you address potential educational topics, it might be helpful to include LGB issues as one topic as well.

Reviewer Expertise:

NA

No decision status is available
